# The Superiority of Large Probes in Treatment of Strictures of the Lachrymal Duct

**Published:** 1887-10

**Authors:** R. O. Cotter

**Affiliations:** Macon, Ga.


### THE SUPERIORITY OF LARGE PROBES IN TREATMENT OF STRICTURES OF THE LACHRYMAL DUCT.

BY R. O. COTTER, M. D., MACON, GA.

Stellwag, in his text-book on eye diseases, speaks of the better effects produced by small probes. I was educated with this belief, but for the past three years noticed that I frequently had cases of lachrymal disease that would not get well after I had properly operated upon the strictures, though I probed them carefully with Bowman's probes at varying intervals, yet the tears would continue to overflow, greatly to the patient's and my own dissatisfaction.

Finally, I read an article upon the use of large aluminium probes by Dr. S. Theobold, of Baltimore. He advocated the use of probes, even up to No. 16. He had designed a set of probes constructed of aluminium, ranging from No. 7 to No. 16, and reported great satisfaction from their use. I had never employed a larger than No. 8. After trying some of his larger sized probes, I can only add my highest indorsement to his plan.

I have used as high as ten in an eight-year-old child, where the trouble had persisted for twelve months after the operation, with the most satisfactory results. So far I have not yet been disappointed in the large probes in any case.

